# Supplementary material for: Comparative metagenomics of tropical reef fishes show conserved core gut functions across hosts and diets with diet-related functional gene enrichments
Source: Appl Environ Microbiol. 2025 Jan 22;91(2):e02229-24. doi: 10.1128/aem.02229-24 (PMC11837501; doi:10.1128/aem.02229-24)
Supplement: Supplemental material — Tables S1 and S2; Figures S1 to S5. [file aem.02229-24-s0001.docx]

**Supplemental Tables and Figures:**

**Supplemental Table 1**: Food analysis between mysis shrimp and fish-meal pellets from NP Analytics Laboratory analyzing protein by combustion, fatty acids by FANL/FANO, fiber, volatile organic acids, and minerals.


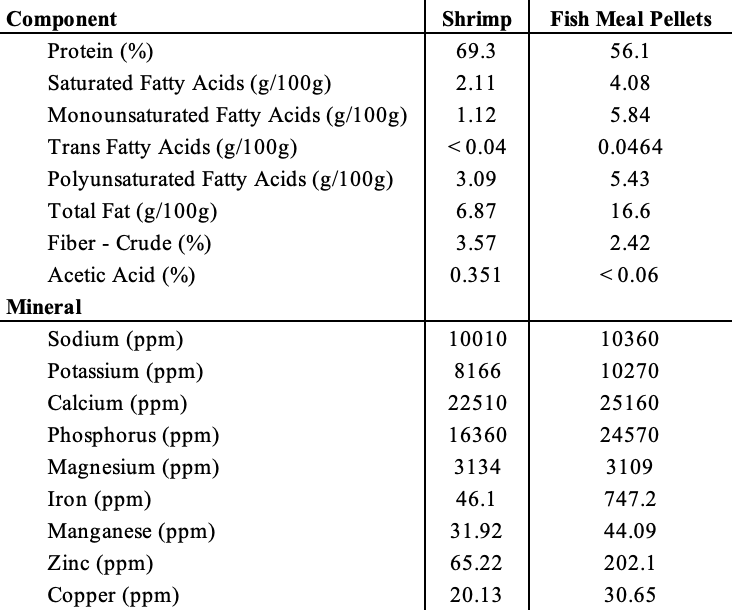


**Supplemental Table 2**: Food compositional analysis between mysis shrimp and fish-meal pellets from NP Analytics Laboratory analyzing fatty acids by FANL/FANO.


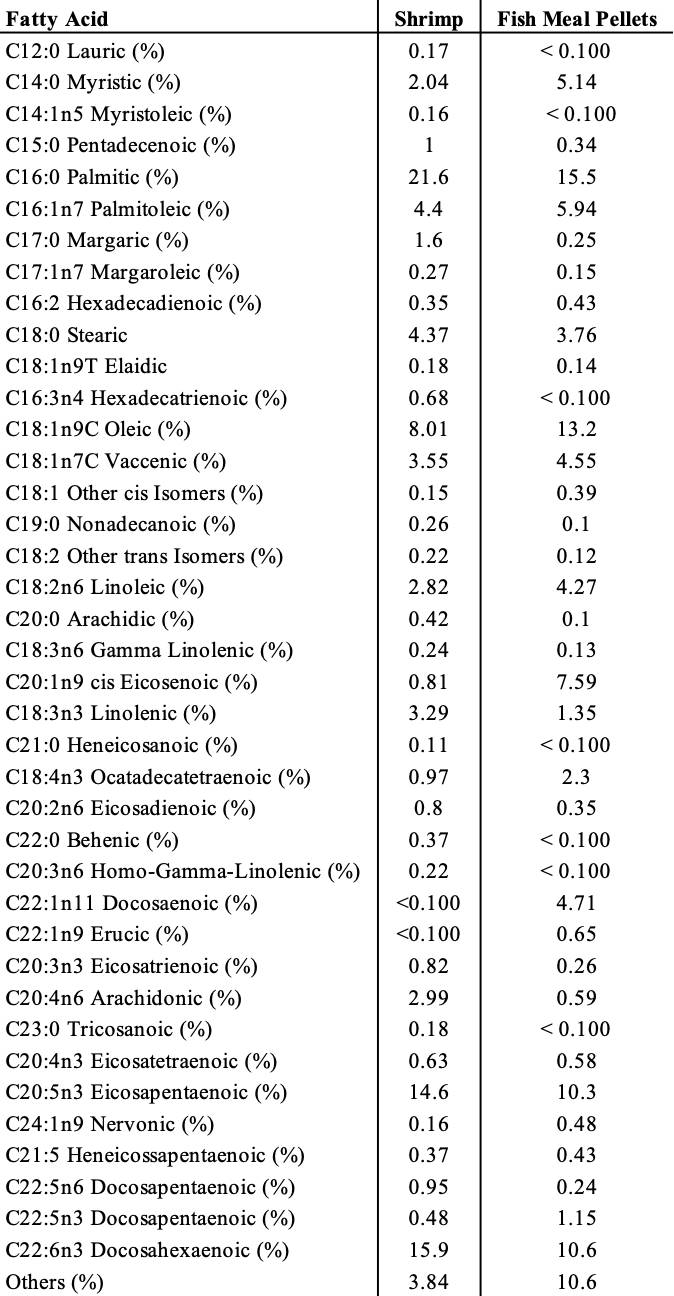


**
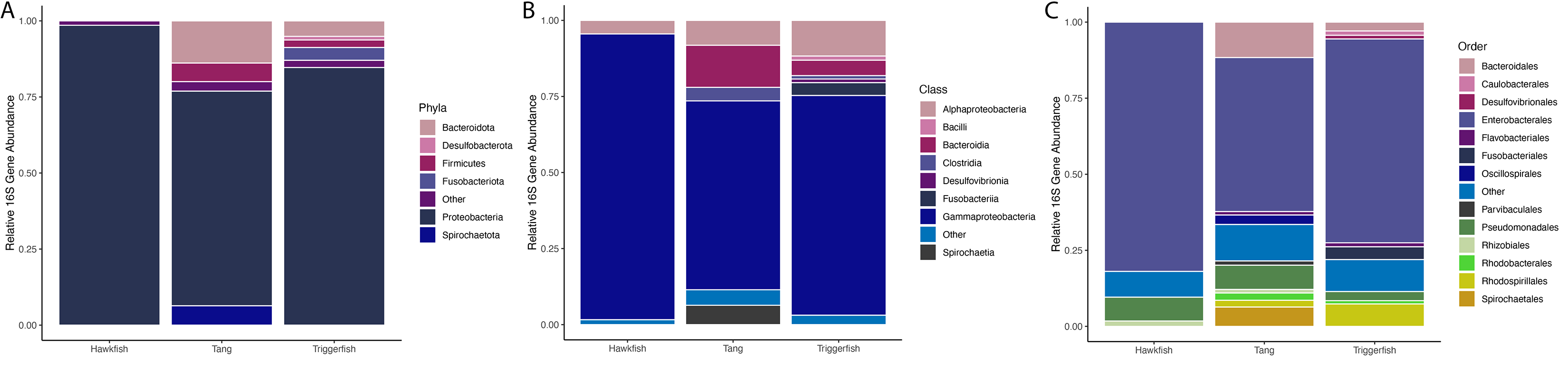
**

**Supplemental Figure 1:** Relative abundance of 16S rRNA genes in each fish listed at the A) Phylum B) Class and C) Order levels.


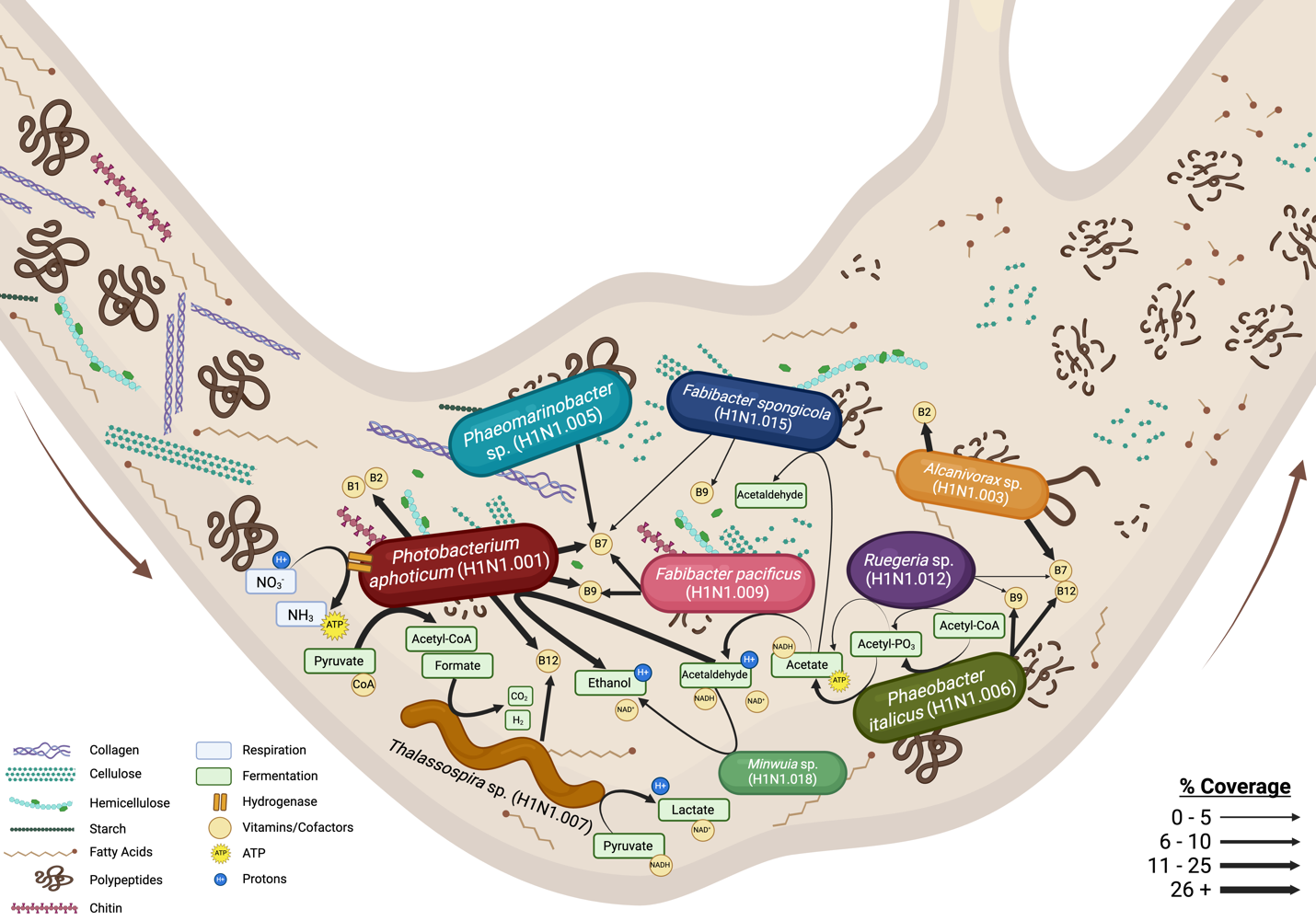


**Supplemental Figure 2:** Diagrammed annotations within the picivorous hawkfish MAGs. Arrows are sized to represent the MAG coverage.

**
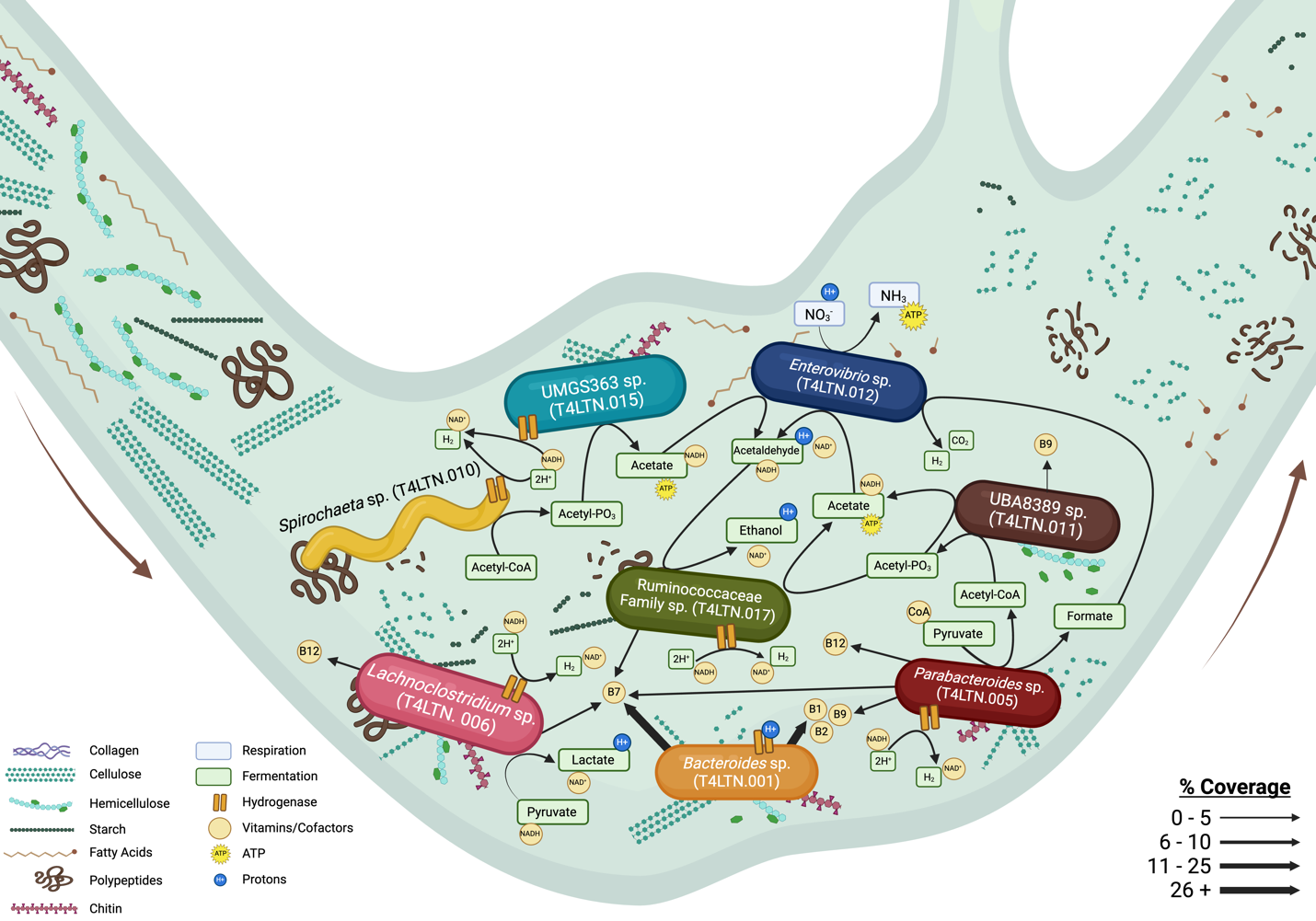
**

**Supplemental Figure 3:** Diagrammed annotations within the herbivorous yellow tang MAGs. Arrows are sized to represent the MAG coverage.


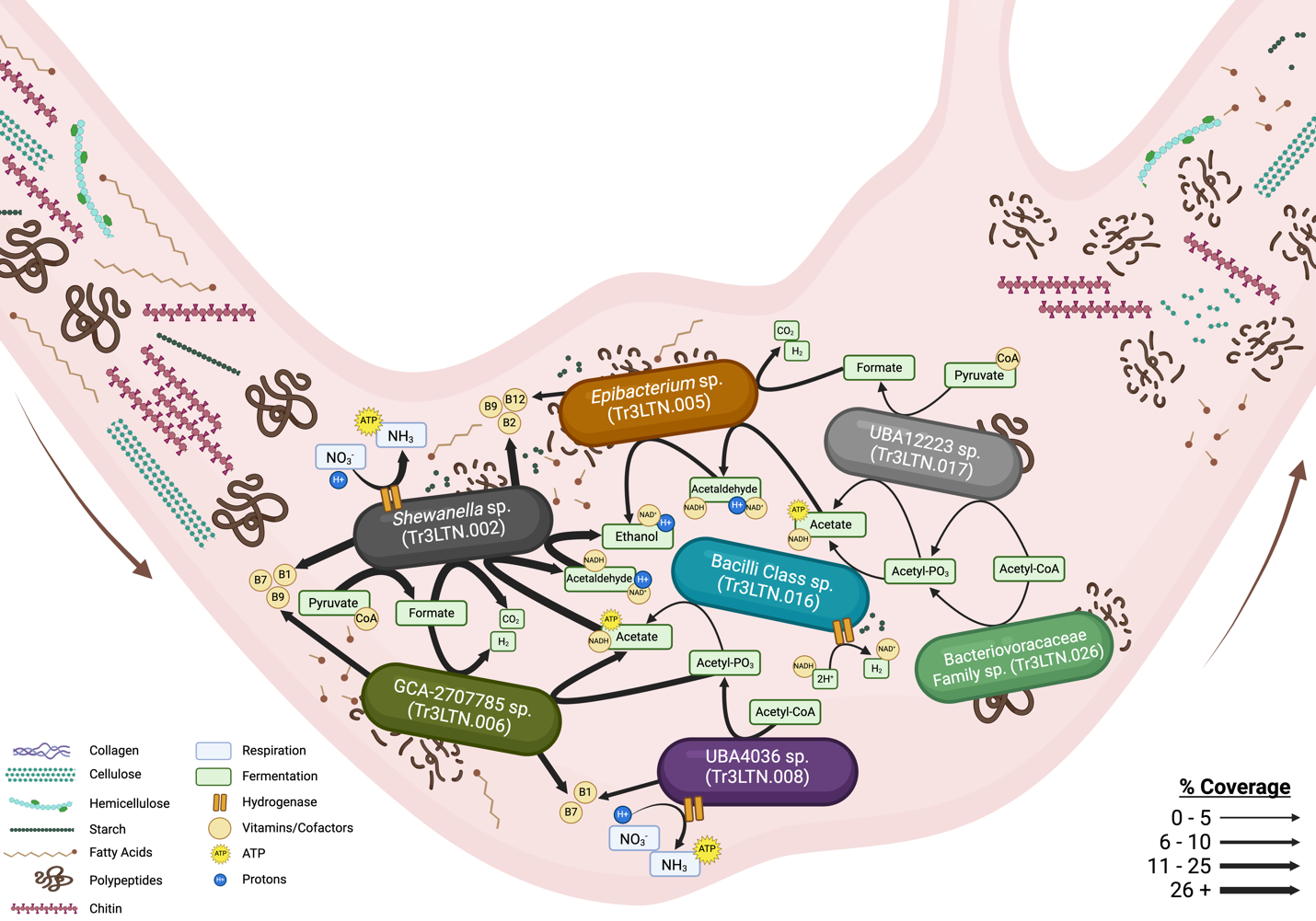


**Supplemental Figure 4:** Diagrammed annotations within the invertivorous triggerfish MAGs. Arrows are sized to represent the MAG coverage.


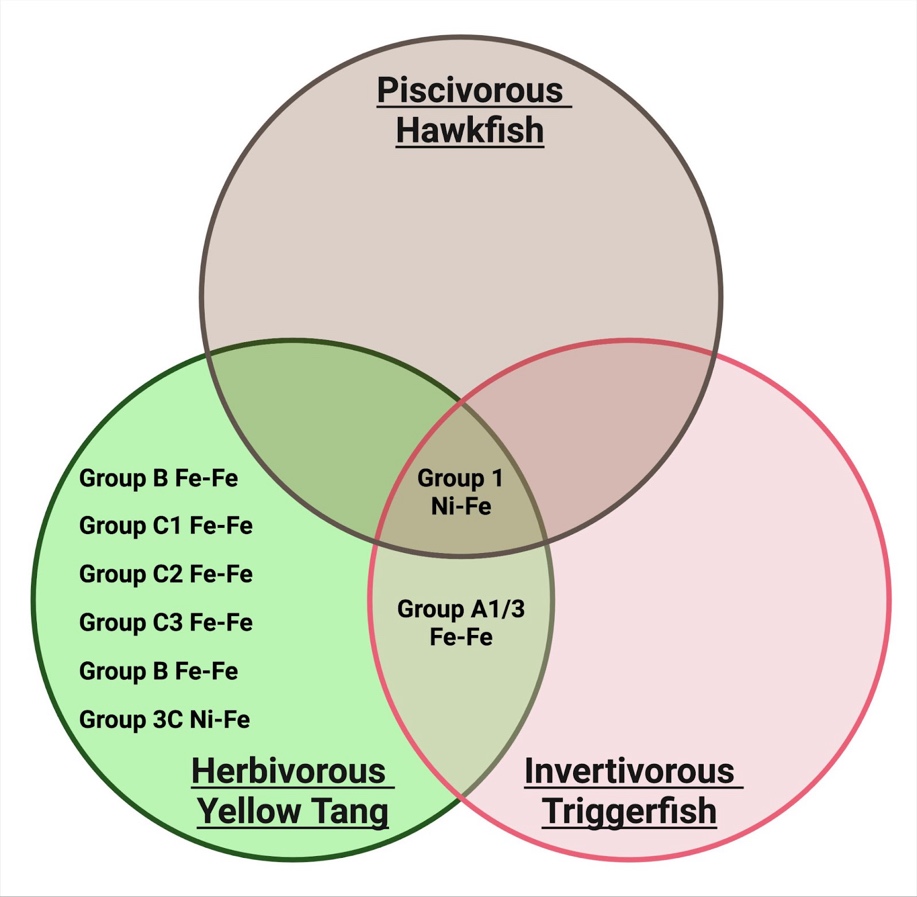


**Supplemental Figure 5**: Hydrogenase groups encoded in MAGs from each gut microbiome.
